# Evaluation of Various State of the Art Head Pose Estimation Algorithms for Clinical Scenarios

**DOI:** 10.3390/s22186850

**Published:** 2022-09-10

**Authors:** Yassine Hammadi, François Grondin, François Ferland, Karina Lebel

**Affiliations:** 1 Department of Electrical and Computer Engineering, Faculty of Engineering, Université de Sherbrooke, Sherbrooke, QC J1H 5N4, Canada; 2 Research Center on Aging, Sherbrooke, QC J1H 4C4, Canada; 3 Interdisciplinary Institute for Technological Innovation (3IT), Université de Sherbrooke, Sherbrooke, QC J1K 0A5, Canada

**Keywords:** movement analysis, face recognition, neural networks, OpenFace 2.0, 3DDFA_V2, realsense, head pose estimation, parkinson’s disease

## Abstract

Head pose assessment can reveal important clinical information on human motor control. Quantitative assessment have the potential to objectively evaluate head pose and movements’ specifics, in order to monitor the progression of a disease or the effectiveness of a treatment. Optoelectronic camera-based motion-capture systems, recognized as a gold standard in clinical biomechanics, have been proposed for head pose estimation. However, these systems require markers to be positioned on the person’s face which is impractical for everyday clinical practice. Furthermore, the limited access to this type of equipment and the emerging trend to assess mobility in natural environments support the development of algorithms capable of estimating head orientation using off-the-shelf sensors, such as RGB cameras. Although artificial vision is a popular field of research, limited validation of human pose estimation based on image recognition suitable for clinical applications has been performed. This paper first provides a brief review of available head pose estimation algorithms in the literature. Current state-of-the-art head pose algorithms designed to capture the facial geometry from videos, OpenFace 2.0, MediaPipe and 3DDFA_V2, are then further evaluated and compared. Accuracy is assessed by comparing both approaches to a baseline, measured with an optoelectronic camera-based motion-capture system. Results reveal a mean error lower or equal to $5.6^{\circ }$ for 3DDFA_V2 depending on the plane of movement, while the mean error reaches $14.1^{\circ }$ and $11.0^{\circ }$ for OpenFace 2.0 and MediaPipe, respectively. This demonstrates the superiority of the 3DDFA_V2 algorithm in estimating head pose, in different directions of motion, and suggests that this algorithm can be used in clinical scenarios.

## 1. Introduction

Head pose analysis plays an important role in recognizing people activity and understanding their interpersonal communication. Head pose estimation (HPE) refers to the analysis of input images or video sequences to identify and characterize the human head’s orientation in 3-dimensional space [1,2,3,4]. HPE remains challenging due to different lighting conditions, occlusions, and various facial expressions that may affect the algorithms’ accuracy [1]. Furthermore, the importance of head orientation relative to the camera field of view may impact the ability of the algorithm to recognize the head properly, and thus estimate its orientation.

Head pose estimation is usually divided in three steps [5]: face detection (i.e., determines if there is a face in the image), face landmarks localisation (i.e., identifies the locations of the facial key landmarks on facial images or videos frames [6]), and head angle estimation (i.e., 3D orientation of the head).

Face detection can be subdivided into two categories: (i) hand-craft based methods and (ii) deep learning methods [5]. Hand-crafted face detection methods have been used extensively in several computer vision applications [7]. These methods include one of the most popular face detection algorithms, called the Viola–Jones face detection method [8], which offers high accuracy when a person faces the camera, but performs poorly in other situations such as self-occlusion [9]. In fact, handcrafted methods highly rely on non-robust features, making the face detection pipeline sub-optimal. While these methods run efficiently on a CPU, they remain sensitive to visual variations of faces [10]. Amongst statistical learning methods, convolutional neural networks (CNN) have been used extensively for face detection [7]. Studies demonstrate that learned features with deep neural networks are robust to the large variation in facial appearances, providing state-of-the-art performance. However, they involve a significant amount of computations, which makes real-time execution challenging, especially on a CPU [10].

For example, Zhang et al. [11] propose a system for frontal face detection using a multi-task cascaded convolutional neural network (MTCNN) approach. The experimental results demonstrate that this technique allows for accurate real-time face detection. However, it is prone to errors from poor head localization. Recently, Zhanga et al. [10] introduced a novel face detector, FaceBoxes, deemed to optimize the speed-accuracy trade-off. FaceBoxes has shown superior performance on both speed and accuracy when evaluated using face detection benchmark datasets including Annotated Faces in the Wild (AFW) [12], PASCAL face [13], Face Detection Data Set and Benchmark (FDDB) [14] and WIDER FACE [15]. Yet, FaceBoxes is limited to face detection and ignores the other steps required for HPE.

As far as facial landmarks estimation is concerned, an early example of a dedicated algorithm is the DLIB toolkit [16], which includes face detection and estimation of 68 landmarks. This method focuses on the front outline of a human face, limiting its capabilities to detect profile or highly occluded faces [16,17]. Other landmarks detectors are designed to solve the problems associated to distortion, posture, and angle. For instance, multi-task cascaded convolutional neural network (MTCNN) [11] uses relatively light CNNs to detect facial regions and extract 5 facial landmark points in real time. This set of points is sufficient to perform alignment, and mitigates the detrimental effects of pose variance. When adding layers to MTCNN, the EMTCNN model [18] can extract 68 facial landmark points in real time. To improve the detection accuracy, Kim et al. [19] suggested a fast and accurate facial landmark detection network using an augmented EMTCNN [18], applying two convolution techniques: dilated convolution and CoordConv [20]. Augmented EMTCNN and EMTCNN extracted 68 landmark points for the 200×200 pixel input images at speeds of 68 FPS (Frames Per Second) and 70 FPS, respectively, using an Intel (R) Core (TM) i7-8700 CPU. Although Augmented EMTCNN is slightly slower than its predecessor, its extraction accuracy almost doubles when compared to EMTCNN, while keeping an extraction speed of 68 FPS, which is sufficient for real-time processing.

At the moment of conducting this study, the source code of Augmented EMTCN approach (https://github.com/guihon12/Augmented_MTCNN (accessed on 1 February 2021)) is no longer available. Recently, Kartynnik et al. [21] proposed the Google MediaPipe library which includes functionalities for facial and body detection and pose analysis. MediaPipe is an open-source, cross-platform approach that allows real-time processing of streaming media such as video and data series. The approach allows detection of up to 468 facial landmarks from images or videos and outputs the coordinates of these landmarks (https://google.github.io/mediapipe/solutions/face_mesh (accessed on 7 July 2022)). The library also offers additional functionalities to process further this information, including pose estimation. MediaPipe library is supported on multiple platforms including Linux, Android, macOS, and Windows.

Once facial landmarks are identified, the last step required for head position estimation consists in assessing the 3D orientation of the head, commonly referred to as yaw, pitch and roll [1] in technical terms or flexion/extension, rotation, and lateral flexion in rehabilitation and clinical terms. Cao et al. [22] proposed the OpenPose toolkit for head and body pose estimation. However, the toolbox is computationally demanding and requires GPU acceleration to achieve real-time performance. Moreover, OpenPose’s face extractor performs poorly on FDDB [14] benchmark dataset because it overestimates the real region of a face and fails to match the oversized bounding boxes to the face annotations (true positives) caused by pose variability [23]. Tadas Baltrusaitis et al. [17] introduced OpenFace 2.0, an extension to the original OpenFace toolkit [24] for facial behavior analysis which includes facial landmarks location, head pose estimation, eye gaze, and facial expressions, all based on Dlib face detector. OpenFace 2.0 has been developed to improve head pose accuracy under varying conditions such as non-frontal or occluded face and under low illumination conditions [17]. On a quad core 3.5 GHz Intel i7-2700K processor with no GPU support, it can estimate face landmark positions at 30 FPS when processing a 640 × 480 pixel video. However, when the angle between the person and the camera becomes too large, an important portion of the face becomes occluded. Thus, some landmarks cannot be associated to the image data, resulting in poor head orientation estimation.

To reduce the impact of self-occlusion and the resulting high non-linearities in head pose estimation for large angles, Zhu et al. [25] proposed 3D Dense Face Alignment (3DDFA). This alignment framework uses a dense 3D face model fitted to the image via a cascaded convolutional neutral network (CNN) based regression method. This method achieves excellent head pose estimation performance on RGB-image-based datasets such as AFLW [26], AFLW2000-3D [25] and 300 W [27]. However, it is demanding in terms of speed and memory allocation [25]. To achieve real-time performance while maintaining high accuracy, Guo et al. [28] introduced 3DDFA_V2, a novel regression framework which offers an interesting speed-accuracy trade-off.

While many head pose estimation approaches have been proposed in the literature, their use in clinical applications or research still remains to be validated [29]. The previously introduced approaches have been evaluated in terms of computational performance, speed, and ability to identify faces. However, no studies, to our knowledge, assess the HPE accuracy in comparison to a gold standard. The present study thus aims at investigating the accuracy of OpenFace 2.0, 3DDFA_V2 and MediaPipe HPE algorithms using video streams against an optoelectronic motion-capture gold standard. The results can provide an important assessment of the suitability of the approaches for clinical use.

There are two main contributions for this paper: (1) we review the state of the art of the existing algorithms for face pose estimation, paired with a discussion on the advantages and disadvantages inherent to each approach and (2) we assess the accuracy of three of the most promising algorithms, OpenFace 2.0, 3DDFA_V2 and Mediapipe, against an optoelectronic baseline. The rest of this paper is organised as follows. Section 2 first compares various head pose estimation algorithms based on their reported characteristics and performance. The validation study methodology, including the experimental setup and the data reduction and analysis follows. Detailed results are then presented in Section 3. Finally, results are discussed in Section 4.

## 2. Materials and Methods

This section compares the available pose estimation algorithms within the literature in terms of data type used, number of landmarks, types of analysis performed and performances. From this analysis, algorithms to be further investigated are selected. The detailed study methodology then follows.

### 2.1. Pose Estimation Algorithms

The number of pose estimation algorithms proposed in the literature has increased considerably in the last few years. Table 1 lists the most refereed algorithms, along with their main characteristics, functionalities and reported performance indices. When assessed, performance was characterized as either using the Mean Normalized Distance (MND) or the Normalized Mean Error (NME), estimated on the 300VW dataset [27]. Lower value of MND or NME are generally regarded as indicators of good precision.

From this analysis, three algorithms stood out for head pose estimation validation: OpenFace 2.0 [17], 3DDFA_V2 [28] and MediaPipe [21]. According to Table 1, OpenFace 2.0 and 3DDFA_V2 both use 68 landmarks to estimate the pose while MediaPipe uses 468 landmarks, covering all face regions and maximize head pose estimation precision. In addition, these methods are freely available to the research community and perform facial landmark detection from images and videos. While EMTCNN also appears interesting, the source code is not publicly available at the time of publication of this paper. Further details on the selected algorithms are given in the following subsections.

#### 2.1.1. OpenFace 2.0

OpenFace 2.0 is a toolbox for facial behavior analysis (facial landmark detection, head pose estimation, eye gaze estimation, and facial action unit recognition) [17]. The source code is available on Github (https://github.com/TadasBaltrusaitis/OpenFace (accessed on 1 June 2020)) and offers possibilities for integration in C++, C#, or Matlab based projects. OpenFace 2.0 can operate on real-time data video feeds from a webcam, recorded video files, image sequences or individual images. Processed data and outputs (facial landmarks, shape parameters, head pose, action units, and gaze vectors) can be saved as CSV files. OpenFace 2.0 uses the recently proposed Convolutional Experts Constrained Local Model (CE-CLM) [30] for facial landmark detection and tracking. The two main components of CE-CLM are: (1) point distribution model (PDM), which captures landmark shape variations, and (2) patch experts, which model local appearance variations of each landmark.

#### 2.1.2. 3DDFA_V2

3DDFA_V2 proposes a new regression framework that makes a reliable balance between accuracy, speed, and stability. This approach runs over 50 FPS on a single CPU core or over 130 FPS on multiple CPU cores (i5-8259U processor) and includes the face detector FaceBoxes instead of Dlib while keeping promising accuracy, and stability [28]. Moreover, it dynamically optimizes the parameters through a novel strategy that combines the fast Weighted Parameter Distance Cost (WPDC) and Vertex Distance Cost (VDC). In addition, using a mini-batch, one still image is smoothly changed into a brief synthetic video including both in-plane and out-of-plane rotations, providing temporal information of adjacent frames for training. The Python source code for this method is available on Github (https://github.com/cleardusk/3DDFA_V2 (accessed on 1 February 2021)) and ready to be used on Gradio (https://gradio.app/hub/AK391/3DDFA_V2 (accessed on 1 February 2021)).

#### 2.1.3. MediaPipe

MediaPipe is based on a deep learning network to provide face and body pose estimation from videos. The model is light enough to be used with portable devices [31]. MediaPipe library contains a general pose estimation model which provides a total of 33 landmarks, 11 of which are used for the face. Yet, the performance of this model is highly variable depending on occlusions (i.e., part of the body is not visible). The MediaPipe FaceMesh model [21] considers a geometric approach to estimate a total of 468 facial landmarks in three dimensions, making the approach more robust to an outlier while computing the homography parameters. Head pose estimation is then performed using a perspective n-points approach.

### 2.2. Experimental Setup

**Figure 1:**
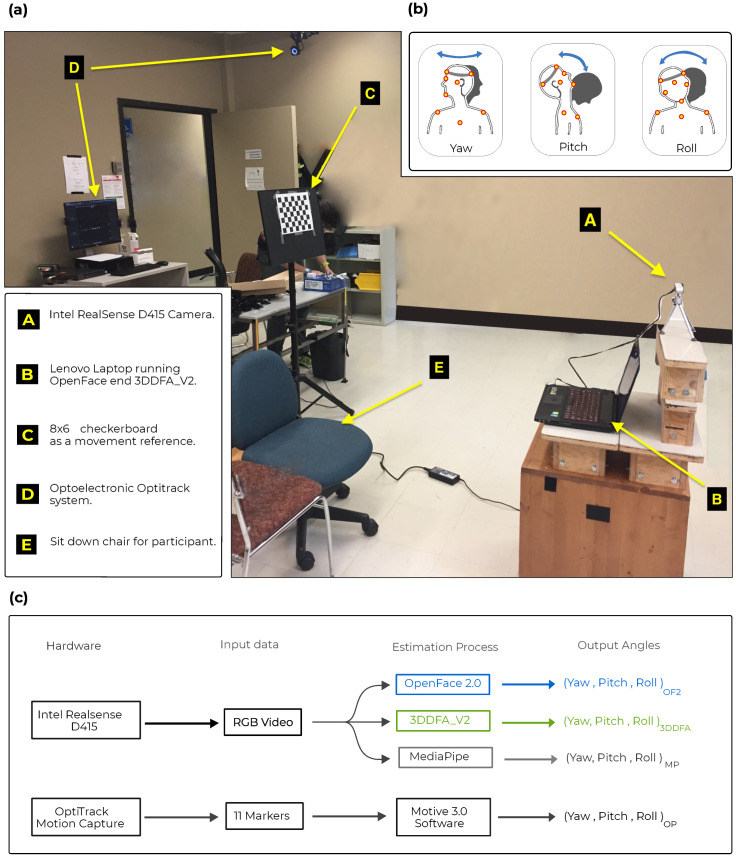
Methodology overview. Panel (**a**) General laboratory setup including the RGB camera in A, the laptop in B, the 8 × 6 checkerboard used to ancre movement into the environment in C and the optoelectronic motion-capture system in D. Panel (**b**) Head orientation movements acquired including head rotation (yaw), flexion-extension (pitch) and lateral flexion (roll). The orange circles illustrate the position of retro-reflective markers. Panel (**c**) Data flow process for both gold standard and camera-based pose estimation algorithms.

A 25-year-old healthy young adult participated in the study. The participant was equipped with 11 markers placed on its head and upper torso, as illustrated in Figure 1. These markers allowed movements to be captured in 3D by a gold standard optoelectronic motion capture system (OptiTrack, NaturalPoint, Corvallis, OR, USA) with eight Prime 13 w cameras. Movements were monitored simultaneously using one Intel RealSense D415 [32] camera positioned 1 m from the participant and oriented towards the face of participant. The camera field of view thus included the head and the trunk.

The participant was instructed to sit on a chair with his head in a neutral, straight-ahead target position, and then to perform a series of pre-defined head movements (see Figure 1b. In total, 24 trials were performed, including a variety of head movements (flexion, extension, rotation to the left/right, right and left lateral flexion) with final orientation aiming towards small (±25) or wide (±60) angles. Calibration was performed to identify the relative position and orientation of the camera at any instant in time with respect to a fixed reference point (8 × 6 chessboard pattern). All tests were processed on a machine running Ubuntu 18.04 OS with a 64-bit Intel Core i7 2.40 GHz CPU and 16 GB memory. Head pose information was extracted using the code or executable provided by the authors of OpenFace 2.0 and 3DDFA_V2 algorithms.

### 2.3. Data Reduction and Analysis

In order to evaluate the accuracy of the head pose algorithms, we analyzed videos for each movement plan, comparing the head estimation angles obtained using the OpenFace 2.0 and 3DDFA_V2 algorithms with those of the Optitrack motion system (gold standard). Synchronicity between the data acquired by both systems was verified using a cross correlation approach.

Equation (1) defines the error in the head pose estimation at the end of the movement in comparison to the value assessed with the gold standard. This metric is used to assess the head pose estimation performance:

(1) $$Error=(\theta_{op\;\max}-{\hat{\theta }}_{k\;\max}),$$

where $\theta_{op\max}$ is the ground-truth maximum head-pose angle calculated with the Optitrack system and ${\hat{\theta }}_{k\max}$ represents the maximum head pose angle estimated by algorithm k∈{OpenFace2.0,3DDFA_2}.

Additionally, the instantaneous absolute error $AE_{f}$ corresponds to the difference between the reference and the estimated head orientation. Analysis of $AE_{f}$ allows to define the stalling angle, corresponding to the moment at which the algorithm fails to estimate the head angle with acceptable performances:

(2) $$AE_{f}=\left|{\left(\theta_{op}\right)}_{f}-{\left({\hat{\theta }}_{k}\right)}_{f}\right|,$$

where ${\left(\theta_{op}\right)}_{f}$ is the ground-truth head-pose angles calculated with Optitrack system at frame *f* and ${\hat{\theta }}_{k}$ stands the estimations angles at frame *f* with the algorithm k∈{OpenFace2.0,3DDFA_2}.

## 3. Results

**Figure 2:**
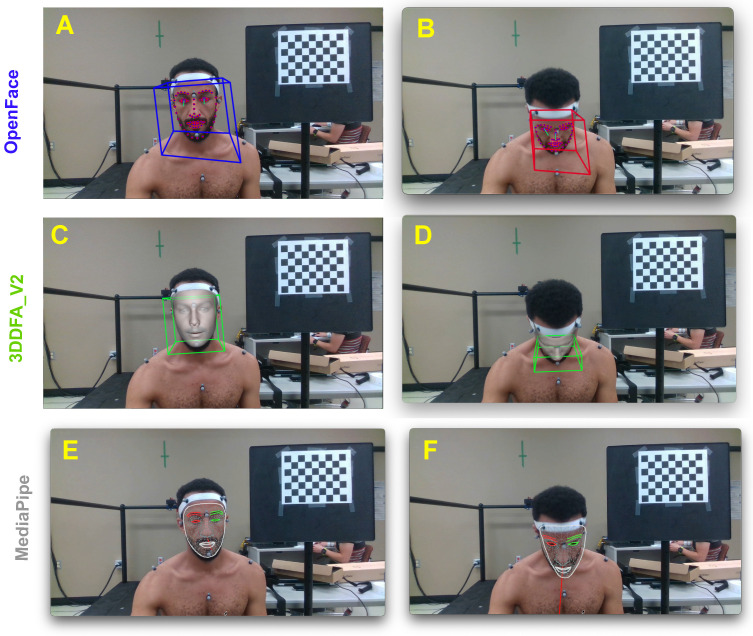
Head movement capture and processing examples. (**A**) Frontal pose estimation with OpenFace 2.0. (**B**) Erroneous pose estimation with OpenFace 2.0 for large pitch angle. (**C**) Frontal pose estimation with 3DDFA_V2. (**D**) Pose estimation with 3DDFA_V2 for large pitch angle. (**E**) Frontal pose estimation with MediaPipe. (**F**) Erroneous pose estimation with MediaPipe at high pitch angles.

A total of 24 video trials were analyzed successfully using OpenFace 2.0, 3DDFA_V2 and MediaPipe. Figure 2 illustrates processing examples of all three algorithms for small and large amplitudes of movement. Table 2 displays the average difference (bias) between the estimated values of the algorithms (OpenFace 2.0, 3DDFA_V2, and MediaPipe) and the reference standard (Optitrack). All approaches estimate head pose on lateral flexion (Roll) within 1.37${}^{\circ }$ (Table 2). However, under large rotations (Yaw) and flexion/extension angles (Pitch), both Openface 2.0 and MediaPipe accuracy reduces (Openface 2.0: 12.37${}^{\circ }$ in Yaw and 14.12${}^{\circ }$ in Pitch; MediaPipe: 11.00${}^{\circ }$ in Yaw and 7.00${}^{\circ }$ in Pitch). In these same conditions, 3DDFA_V2 reports a higher mean accuracy of 5.62${}^{\circ }$ in Yaw and 0.87${}^{\circ }$ in Pitch.

**Figure 3:**
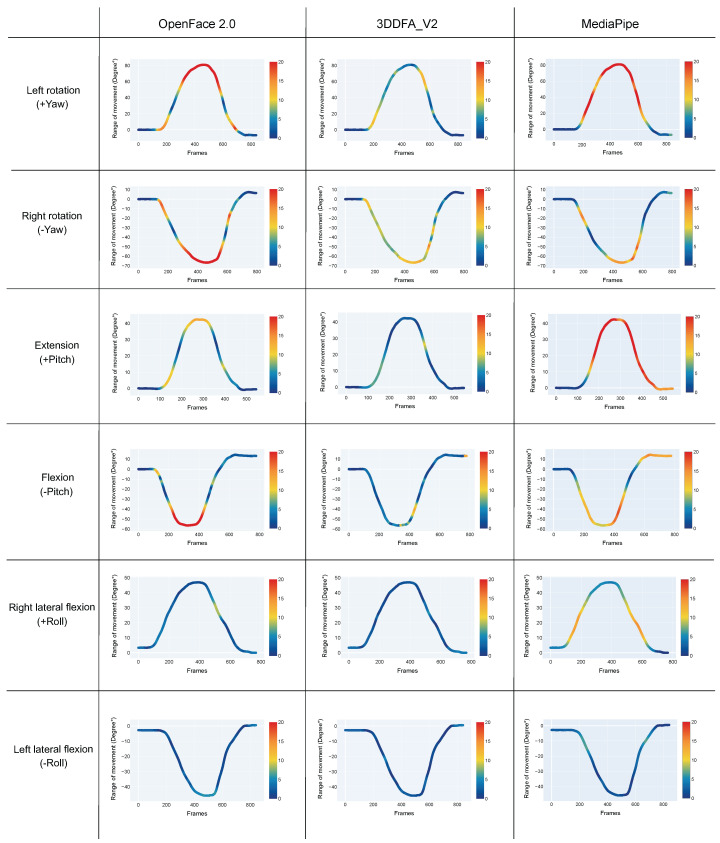
Accuracy variation results during head movements. Each panel represents the movement captured by the gold standard, per plane of motion. The absolute error assessed by each method is shown with the color of the curve: dark blue corresponds to a 0${}^{\circ }$ error, dark red to an error greater or equal to 20${}^{\circ }$.

To further analyze the impact of the range and plane of movement on pose estimation algorithms’ accuracy, the orientation estimation error is considered for each frame. Figure 3 shows the variation in absolute error for each plane and direction of movement.

Assuming a tolerable error rate of 10${}^{\circ }$, Table 3 shows that MediaPipe has a limitation of $\approx 35^{\circ }$ in flexion/extension, which limitation increases to $\approx 40^{\circ }$ for OpenPose 2.0. 3DDFA_V2 performs within the tolerable error rate with movements in flexion/extension up to 58${}^{\circ }$ (as demonstrated in Figure 3). Rotation movements were assessed within tolerance throughout the range of motion with 3DDFA_V2. However, error unexpectedly increased to 26${}^{\circ }$ with OpenFace 2.0 when rotation exceeded 40${}^{\circ }$. Similar behaviour occurred with MediaPipe when rotation reached 30${}^{\circ }$. This failure is mainly caused by the inaccuracy of facial landmarks detection (Figure 3). Errors in lateral flexion (Roll), remained within 5${}^{\circ }$ for all three algorithms even at large angles.

## 4. Discussion

This study assessed the accuracy of OpenFace 2.0, 3DDFA_V2 and MediaPipe, three methods to estimate head pose from a video, using an optoelectronic camera system as a gold standard. The results obtained in this study demonstrate that the 3DDFA_V2 method is more robust than both OpenFace 2.0 and MediaPipe to capture head pose. More specifically, the average error obtained with 3DDFA_V2 remained below or close to an acceptable level of 5${}^{\circ }$ degrees regardless of the plane of motion. Langland et al. [33] agreed that an error of approximately 5${}^{\circ }$ is deemed negligible for most practical clinical purposes. Accordingly, 3DDFA_V2 appears to be suitable for clinical applications. On the other hand, OpenFace 2.0’s accuracy varied between 1${}^{\circ }$ and 14${}^{\circ }$ depending on the type of movement performed. Similarly, MediaPipe revealed an accuracy varying between 1${}^{\circ }$ and 11${}^{\circ }$. Yet, analysis of the stalling angle reveals that both OpenFace 2.0 and MediaPipe can be used under constrained conditions such as to capture small amplitude movements performed mainly while looking towards the camera.

In clinical context where accuracy of the measurement is important, the advantages of 3DDFA_V2 are significant. This method can provide clinicians with additional useful and reliable information to monitor the patient’s condition and progression. On a clinical usability point of view, approaches based on RGB cameras also have major advantages compared to optoelectronic systems: they are easier to setup, cost less, and they work without markers. Markers not only increase setup time, but they may impact patients’ movements due to altered proprioception. The approach proposed in this study is based on RGB video collected using an off-the-shelf camera and processed using open source algorithms. This setup offers a potentially inexpensive, portable and convenient tool to allow clinicians to obtain accurate, reliable and objective readings. So far, technology has to be properly implemented in a clinical tool and validated in order to reach its full potential.

Recently, Zhang et al. [34] demonstrated the clinical potential of using a video-based approach to capture severity of cervical dystonia. Overall, they demonstrated a good correlation between the video-based algorithm and the clinically validated Toronto Western Systemic Torticolis Rating Scale version 2. Yet, they reported high variations in correlation in pitch, which may have altered the overall results. Considering that the videos were processed using OpenPose 2.0 with the now known limitations in accuracy, this tends to corroborate the clinical potential of a clinical tool based on 3DDFA_V2.

## 5. Conclusions

This study shows that head pose estimation by means of a RGB camera has an excellent agreement with a reference optoelectronic gold standard. Specifically, the 3DDFA_V2 method achieved accurate and robust head pose estimation in various conditions of movement and showed only minimal deviations compared with both OpenFace 2.0 and MediaPipe. The next logical step will be to validate 3DDFA_V2 algorithms under true clinical conditions, with participants living with physical limitations, in order to assess the clinical advantages of such technologies to support clinicians in their diagnosis.

## Figures and Tables

**Table 1:** Head pose estimation algorithms benchmarking. Performances are evaluated in terms of Frames Per Second (FPS), Mean Normalized Distance (MND) and Normalized Mean Error (NME).

Algorithm	Landmarks				Facial Analysis Tasks				Performances			Availability of the Source Code
	2D	3D	Number		Pose	Expression	Gaze		FPS	MND	NME	
Dlib [16]	✓	✗	68		✗	✗	✗		15	-	-	✓
OpenPose [22]	✓	✓	70		✓	✗	✗		22	-	-	✓
OpenFace [24]	✓	✓	68		✓	✓	✓		30	-	-	✓
OpenFace 2.0 [17]	✓	✓	68		✓	✓	✓		30	-	-	✓
MTCNN [11]	✓	✓	5		✓	✗	✗		99	-	-	✓
EMTCNN [18]	✓	✓	68		✓	✗	✗		70	6.63	-	✗
Augmented EMTCNN [19]	✓	✓	68		✓	✗	✗		68	5.59	-	✗
3DDFA [25]	✓	✓	68		✓	✗	✗		20	-	5.42	✓
3DDFA_V2 [28]	✓	✓	68		✓	✗	✗		50	-	3.51	✓
MediaPipe [21]	✓	✓	468		✓	✓	✓		-	-	-	✓

Note. ✓ corresponds to available feature. ✗ stands for missing feature.

**Table 2:** Average errors on head pose estimation for OpenFace 2.0 and 3DDFA_V2.

Algorithms	OpenFace 2.0				3DDFA_V2				MediaPipe		
	**Yaw**	**Pitch**	**Roll**		**Yaw**	**Pitch**	**Roll**		**Yaw**	**Pitch**	**Roll**
**Error (${}^{\circ }$)**	12.37	14.12	−0.75		−5.62	0.87	−0.37		11.00	7.00	1.37
SD (${}^{\circ }$) *	12.30	13.62	2.65		3.33	3.83	3.11		10.65	10.22	2.44

* Standard Deviation.

**Table 3:** Stalling angle per plane of motion for OpenFace 2.0, 3DDFA_V2 and MediaPipe.

		Tolerable Error (5${}^{\circ }$)		Tolerable Error (10${}^{\circ }$)	
**Algorithms**	**Angle**	**Stalling Angle (${}^{\circ }$)**	**SDV**	**Stalling Angle (${}^{\circ }$)**	**SDV**
OpenFace 2.0	+Yaw	32.32	12.14	40.13	3.33
	−Yaw	−30.48	3.14	−50.36	16.52
	+Pitch	33.99	4.70	40.33	3.14
	−Pitch	−41.70	4.28	−57.84	2.27
	+Roll	- *	- *	- *	- *
	−Roll	- *	- *	- *	- *
3DDFA_V2	+Yaw	- *	- *	- *	- *
	−Yaw	−42.6	6.43	−54.24	7.45
	+Pitch	42.7	0.00	57.84	2.27
	−Pitch	−41.09	16.66	- *	- *
	+Roll	- *	- *	- *	- *
	−Roll	- *	- *	- *	- *
MediaPipe	+Yaw	29.18	4.82	29.18	4.82
	−Yaw	−49.48	3.18	−54.44	10.20
	+Pitch	34.00	4.70	34.00	4.70
	−Pitch	−37.80	14.64	- *	- *
	+Roll	- *	- *	- *	- *
	−Roll	- *	- *	- *	- *

* No limitations reached.

## Abbreviations

The following abbreviations are used in this manuscript:

3DDFA_V2	3D Dense Face Alignment Version 2
MTCNN	Multi-Task cascaded Convolutional Neural Network
